# Identification of a neutralizing epitope within minor repeat region of *Plasmodium falciparum* CS protein

**DOI:** 10.1038/s41541-020-00272-6

**Published:** 2021-01-18

**Authors:** J. Mauricio Calvo-Calle, Robert Mitchell, Rita Altszuler, Caroline Othoro, Elizabeth Nardin

**Affiliations:** 1 Department of Microbiology, New York University School of Medicine, New York, NY USA; 2 Present Address: Department of Pathology, University of Massachusetts Medical School, Worcester, MA USA; 3 Present Address: ISGlobal, Hospital Clinic—Universitat de Barcelona, Barcelona, Catalonia Spain

**Keywords:** Adaptive immunity, Vaccines, Malaria

## Abstract

Malaria remains a major cause of morbidity and mortality worldwide with 219 million infections and 435,000 deaths predominantly in Africa. The infective *Plasmodium* sporozoite is the target of a potent humoral immune response that can protect murine, simian and human hosts against challenge by malaria-infected mosquitoes. Early murine studies demonstrated that sporozoites or subunit vaccines based on the sporozoite major surface antigen, the circumsporozoite (CS) protein, elicit antibodies that primarily target the central repeat region of the CS protein. In the current murine studies, using monoclonal antibodies and polyclonal sera obtained following immunization with *P. falciparum* sporozoites or synthetic repeat peptides, we demonstrate differences in the ability of these antibodies to recognize the major and minor repeats contained in the central repeat region. The biological relevance of these differences in fine specificity was explored using a transgenic *P. berghei* rodent parasite expressing the *P. falciparum* CS repeat region. In these in vitro and in vivo studies, we demonstrate that the minor repeat region, comprised of three copies of alternating NANP and NVDP tetramer repeats, contains an epitope recognized by sporozoite-neutralizing antibodies. In contrast, murine monoclonal antibodies specific for the major CS repeats (NANP)_n_ could be isolated from peptide-immunized mice that had limited or no sporozoite-neutralizing activity. These studies highlight the importance of assessing the fine specificity and functions of antirepeat antibodies elicited by *P. falciparum* CS-based vaccines and suggest that the design of immunogens to increase antibody responses to minor CS repeats may enhance vaccine efficacy.

## Introduction

The repeat region of the circumsporozoite (CS) protein of all *Plasmodium* species contains a species-specific immunodominant B cell epitope that is recognized by sera of sporozoite-immunized rodents, monkeys and human volunteers, as well as by naturally infected individuals^1,2^. When sporozoites are targeted by antirepeat antibodies, parasite motility is inhibited thus blocking egress from the site of the mosquito bite into the blood and invasion of host cell hepatocytes thereby preventing subsequent development of blood-stage infection and clinical disease^3–6^.

Early studies demonstrated that monoclonal antibody (MAB) specific for CS repeats derived from sporozoite-immunized experimental hosts were protective following passive transfer to susceptible rodents or monkeys. As little as 10 µg of MAB specific for the CS repeats of rodent *P. berghei* sporozoites was shown to protect naïve rodents against sporozoite challenge and 2 mg of MAB specific for the *P. vivax* CS repeats protected Saimiri monkeys from homologous sporozoite challenge^7^. In recent studies, passive transfer of 100–300 µg of human MAB specific for *P. falciparum* CS repeats, derived from volunteers immunized with the CS-based RTS,S vaccine, protected mice against challenge with transgenic *P. berghei* parasites expressing full length *P. falciparum* CS protein^8^.

**Fig. 1:**
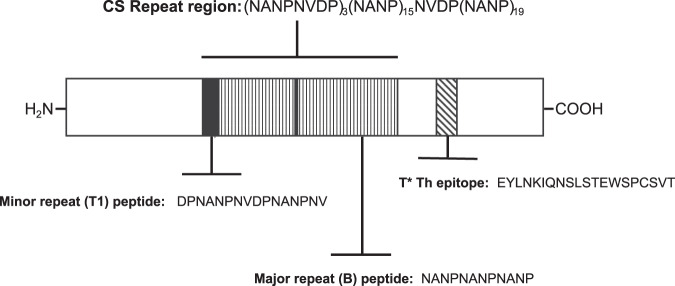
Schematic of the *P. falciparum* CS proteins and peptides. *Illustration of P. falciparum* CS protein showing the B major (NANP) tetramer repeats (open bars) and the T1 minor repeat epitope comprised of alternating NVDP NANP repeats (solid bars). Synthetic tetrabranched peptides representing the major repeats NANPNANPNANP, or minor repeats DPNANPNVDPNANPNV, were used as antigens in the ELISA to measure fine specificity of polyclonal and monoclonal antirepeat antibody responses. The T* epitope (hatched bar) is a universal T helper cell epitope located in the C -terminus (aa 326–345 NF54 isolate) that is recognized by a broad range of human and murine class II HLA genotypes. Peptide immunogens were constructed as either linear or tetrabranched peptides comprised of major repeats combined with the T* universal T helper cell epitope, (NANP)_3_EYLNKIQNSLSTEWSPCSVT (designated BT*), or minor repeats combined with T* universal T helper cell epitope (DPNANPNV)_2_EYLNKIQNSLSTEWSPCSVT (designated T1T*).

The demonstration of antibody-mediated protection in experimental hosts has encouraged efforts to design CS-based vaccines that elicit high titer antirepeat antibodies to neutralize sporozoite infectivity. However, despite decades of vaccine research, a protective antirepeat antibody titer remains to be defined and there is limited information on the range of functional antirepeat antibodies elicited following immunization with sporozoites or CS subunit vaccines. The immunodominant repeat region of *P. falciparum* CS protein is comprised of major and minor tetramer repeat sequences that are conserved in all isolates. In the *P. falciparum* NF54 strain^9^, used in the majority of human malaria challenge studies, the CS repeat region is comprised primarily of 37 NANP tetramers (Fig. 1). In addition, there is a minor repeat region comprised of three alternating NVDP and NANP tetramers in the 5’ repeat region, which is adjacent to a CS protein proteolytic cleavage site that plays a role in sporozoite invasion of host cells^10,11^.

Early studies demonstrated that a major repeat peptide, comprised of three copies of the NANP repeat, (NANP)_3,_ could block binding of monoclonal and polyclonal antirepeat antibodies to *P. falciparum* sporozoites and *P. falciparum* CS protein^12^. The NANP repeats were used in the first CS-based subunit vaccine clinical trials, which tested safety and efficacy of a synthetic peptide-protein conjugate, (NANP)_3_-TT^13^, or a recombinant protein R32 containing 32 NANP repeats^14^. The Phase II trials demonstrated modest protective efficacy following challenge of a small number of immunized volunteers by bites of *P. falciparum*-infected mosquitoes.

Differences in fine specificity of polyclonal antirepeat antibodies elicited in NANP peptide-immunized simian and rodent hosts were noted in early studies based on peptide ELISA and IFA reactivity with *P. falciparum* sporozoites^15,16^. The functional relevance of the variations in fine specificity of antirepeat antibodies could not be defined in these early studies as *P. falciparum* sporozoites are infective only in human hosts or splenectomized chimpanzees. More recently, the development of transgenic rodent parasites expressing all or parts of the *P. falciparum* CS protein has provided rodent models to address the role of antibody fine specificity in neutralizing sporozoite infectivity.

In the current paper, we used transgenic *P. berghei* sporozoites expressing *P. falciparum* CS repeats^17^ to examine neutralizing activity of MAB and polyclonal antirepeat antibodies derived from sporozoite- or peptide-immunized mice. We found that MAB specific for the minor repeats were protective in vitro and in vivo. In addition, several MABs elicited by immunization with major repeats could be isolated, which did not have neutralizing activity. These studies on fine specificity of sporozoite-neutralizing antibodies have important implications for vaccine design and analysis of functional humoral responses to the *P. falciparum* CS repeat region.

## Results

### Fine specificity of MAB derived from mice immunized with *P. falciparum* sporozoites

**Fig. 2:**
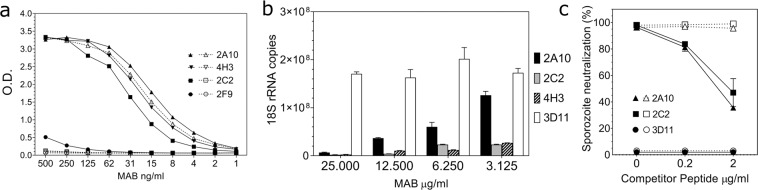
Fine specificity and sporozoite-neutralizing activity of MAB derived from *P. falciparum* sporozoite-immunized mice. **a** MAB initially selected by *P. falciparum* sporozoite IFA were analyzed for fine specificity using CS repeat peptide ELISA. ELISA plates coated with either minor repeat peptide (DPNANPNV)_2_ [closed symbols] or major repeat peptides (NANP)_3_ [open symbols] were reacted with two-fold dilutions of MAB. **b** Sporozoite-neutralizing activity of MAB specific for minor repeats was determined by TSNA using transgenic *P. berghei* sporozoites expressing *P. falciparum* CS repeats (PfPb). Sporozoites were incubated with MAB 2C2 and 4H3, specific for *P. falciparum* CS minor repeats, or MAB 2A10 which cross-reacts with both the minor and major repeats. MAB 3D11 specific for *P. berghei* CS repeats served as negative control. Hepatoma cell extracts of duplicate wells obtained 48 h post sporozoite infection were pooled and tested in triplicate by qRT-PCR and results are shown as mean ± SD 18 S rRNA copy number. At all concentrations tested, the minor repeat-specific MABs 2C2 and 4H3, and MAB 2A10, significantly reduced *Plasmodium* rRNA copy numbers compared to negative control MAB 3D11 (one-way ANOVA with Dunnett’s test, adjusted *P* < 0.0001). **c** A Peptide Competition TSNA was carried out by preincubating 25 µg/ml MAB 2C2 or MAB 2A10 with various concentration of minor repeat peptide (DPNANPNV)_2_ [closed symbols] or major repeat peptide (NANP)_3_ [open symbols]. MAB 3D11 served as negative control. Following incubation of MABs with competitor peptide for 1 h at 37 °C, PfPb sporozoites were added and TSNA was carried out per protocol using qRT-PCR. Results are shown as percent inhibition ± competitor peptide. For MAB 2A10 and MAB 2C2 there was a significant difference in rRNA copy number in presence of 2 µg/ml and 0.2 µg competitor minor repeat peptide versus no peptide (one-way ANOVA with Dunnett’s test, adjusted *P* < 0.0004).

CS-specific MABs derived from *P. falciparum* sporozoite-immunized mice^18^ were analysed for fine specificity by ELISA using either the major repeats (NANP)_3_ or minor repeats (DPNANPNV)_2_ as coating antigen. MAB fine specificity varied, either cross-reacting with both the minor and major repeats, as exemplified by MAB 2A10, or skewing toward the minor repeat region with little or no reactivity with the major repeats as shown by MAB 2F9, 2C2 and 4H3 (Fig. 2a). All of the MABs specific for minor repeats also reacted with *P. falciparum* sporozoites by indirect immunofluorescence assay (IFA) (data not shown).

MABs recognizing only the minor repeats were compared to cross-reacting MAB 2A10 using a Transgenic Sporozoite Neutralizing Assay (TSNA) to measure ability of the MAB to inhibit invasion of PfPb sporozoites into hepatoma cells (Fig. 2b). The minor repeat-specific MABs 2C2 and 4H3 demonstrated dose-dependent sporozoite-neutralizing activity. These MAB gave >90% reduction in parasite rRNA copy number when tested at 25 and 12.5 µg/ml, similar to inhibition obtained with MAB 2A10 that cross-reacted with both major and minor repeats. The minor repeat-specific MABs were also inhibitory at 3.125 µg/ml, the lowest concentration tested. MAB 3D11, specific for *P. berghei* CS repeats, did not block PfPb sporozoite invasion of hepatoma cells. These results provide evidence that antibodies specific for the *P. falciparum* CS minor repeats can neutralize sporozoite infectivity.

To confirm the specificity of neutralizing activity, a peptide competition TSNA was carried out by preincubating minor repeat-specific MAB 2C2, or cross-reacting MAB 2A10, with either major or minor repeat peptides prior to addition to PfPb sporozoites (Fig. 2c). MAB 2C2 was inhibited by minor repeat peptide (closed squares), but not the major repeat peptide (open squares), confirming that antibody neutralizing activity was directed at the minor repeats. Interestingly, MAB 2A10, which recognizes both major and minor repeats in peptide ELISA, was primarily inhibited by the minor repeat peptide. Preincubation of MAB 2A10 with the major repeat peptide gave minimal inhibition of sporozoite neutralization. The peptide competition TSNA confirms that the CS minor repeats are targets of neutralizing antibodies.

### Major or minor repeat peptides elicit antibodies that differ in fine specificity

To further investigate fine specificity of neutralizing antibodies, mice were immunized with tetrabranched peptides containing either the minor repeats (T1T*)_4_ or the major repeats (BT*)_4_. Previous studies had found that a related immunogen (T1BT*)_4_ elicited antirepeat antibodies in mice and human volunteers^19,20^.

**Fig. 3:**
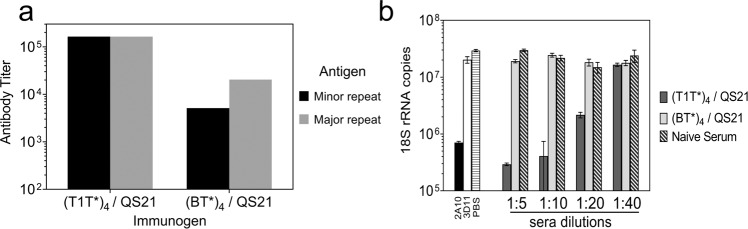
Tetrabranched peptides containing major or minor repeats elicit antibodies that differ in sporozoite-neutralizing activity. C57BL/6 mice were immunized with three doses of tetrabranched peptide containing minor repeats, (T1T*)_4_, or major repeats, (BT*)_4_, using QS21 as adjuvant. **a** IgG ELISA titers against minor repeat peptide (black bars) or major repeat peptide (grey bars) were determined using serum obtained post 3rd dose. Endpoints were taken as final dilution of pooled serum giving OD in peptide-coated wells >3× OD of BSA-coated wells. **b** TSNA was carried out using 1:5–1:40 dilution of pooled serum from mice immunized with minor repeat peptides (black bars) or major repeat peptides (grey bars). MAB 2A10 and MAB 3D11 (25 µg/ml) served as positive and negative controls, respectively. Results are shown as 18 S rRNA copy number mean ± SD measured by qRT-PCR. Inhibition by (T1T*)_4_ immune sera was significantly greater than (BT*)_4_ antisera at 1:5, 1:10 or 1:20 dilution (one-way ANOVA with Tukey’s test, adjusted *P* = 0.0004).

Mice were immunized subcutaneously (s.c.) with three doses of (T1T*)_4_ or (BT*)_4_ in QS21 adjuvant. Immune sera of C57BL/6 mice immunized with (T1T*)_4_ reacted equally with minor repeat peptide (DPNANPNV)_2_ and with major repeat peptide (NANP)_3_ in ELISA (Fig. 3a). In contrast, immune sera of mice immunized with major repeat peptide (BT*)_4_ recognized the major repeats with four-fold higher titer than the minor repeat peptide. Skewing of the fine specificity of the antirepeat antibody response was also observed in BALB/c mice immunized with (T1T*)_4_ or (BT*)_4_ (Supplementary Table 1). The BALB/c mice immunized with (T1T*)_4_ developed eight-fold higher titer against T1 minor repeats compared to major repeats while immunization with (BT*)_4_ elicited eight-fold higher titer against B major repeats.

To examine the function of the antirepeat antibodies elicited by minor repeat peptide, the serum from (T1T*)_4_ immunized C57BL/6 mice was tested for ability to neutralize PfPb sporozoite infectivity in vitro. Sera of BALB/c mice were not analyzed in TSNA due to nonspecific resistance to *P. berghei* that comprise the genetic backbone of the PfPb transgenic sporozoites^21,22^.

In the TSNA, the sera of C57BL/6 mice immunized with (T1T*)_4_ had high levels of neutralizing activity, with a 1:10 serum dilution inhibiting >90% sporozoite invasion of hepatoma cells (Fig. 3b**)**. Inhibition by the (T1T*)_4_ immune serum was comparable to that observed with 25 µg/ml MAB 2A10. The neutralizing activity in (T1T*)_4_ serum was specific for CS repeats as no inhibition was obtained with serum obtained prior to immunization (hatched bars), which gave 18 S rRNA copy number similar to control wells without serum (PBS, horizontal hatched bar). In contrast, serum of C57BL/6 immunized with the major repeat peptide, (BT*)_4_, did not inhibit sporozoite infectivity at the lowest dilution tested (1:5). Since previous studies have demonstrated that high levels of antibodies elicited by (NANP)n constucts can inhibit *P. falciparum* sporozoites^12–14,23^, the lack of neutralizing activity of (BT*)_4_ serum suggests that magnitude, as well as specificity of the antirepeat antibodies, is important in neutralizing sporozoite infectivity.

### Linear peptide containing minor repeats also elicits sporozoite-neutralizing antibodies

To determine if differences in the fine specificity of the antirepeat antibodies were dependent on peptide configuration, mice were immunized with linear T1T* peptides using various adjuvant formulations. Previous murine studies had found that immunogenicity of related T1BT* sequence was comparable using either branched or linear peptides as immunogens^24^. Following three s.c. injections of linear T1T* peptide in various adjuvants in C57BL/6 and BALB/c mice, the linear T1T* peptide was found to elicit antirepeat ELISA titers comparable in magnitude to those elicited by the more complex tetrabranched (T1T*)_4_ (Fig. 3a, Supplementary Fig. 1). Linear peptides formulated in oil-in-water adjuvants, Montanide ISA 720 or ISA 51, or by the inclusion of the TLR 9 agonist, CpG, elicited ELISA titers that were an order of magnitude higher than peptide without adjuvant (PBS). With all adjuvant formulations, immune sera IFA titers with *P. falciparum* sporozoites were similar in magnitude to the repeat ELISA titer.

In a second experiment, a head-to-head comparison of tetrabranched (T1T*)_4_ peptide versus linear T1T* peptide formulated in ISA 720 adjuvant was shown to elicit comparable antirepeat antibodies responses with ELISA GMT 1.6 × 10^4^ and 5.6 × 10^4^, respectively. TSNA demonstrated that immune sera from C57BL/6 mice immunized with either linear T1T* peptide or with (T1T*)_4_ peptide significantly decreased parasite rRNA copy numbers when compared to Day 0 sera (adjusted *P* = 0.0024 and *P* = 0.042, respectively) (Supplementary Fig. 2).

**Fig. 4:**
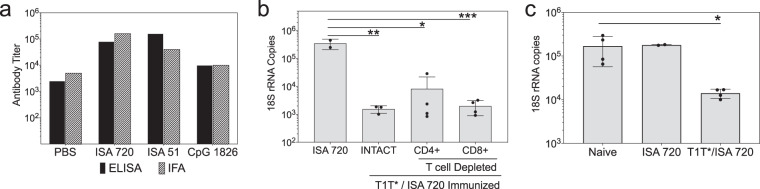
Linear peptides containing minor repeats elicit sporozoite-neutralizing antibodies. **a** C57BL/6 mice were immunized with three doses of linear T1T* peptide without adjuvant (PBS), or with adjuvant comprised of oil-in-water emulsions (ISA 720, ISA 51) or a TLR 9 agonist CpG. Pooled sera were tested against repeat peptide by ELISA or against *P. falciparum* sporozoites by IFA. **b** Mice immunized with three doses of T1T*/ISA 720 were challenged by exposure to the bites of PfPb-infected mosquitoes. A subset of immunized mice were treated with MAB specific for CD4 + or CD8 + T cells prior to challenge. Parasite 18 S rRNA copy numbers in liver extracts obtained 40 h post-challenge were measured by qRT-PCR. Bars show 18 S rRNA copy number mean ± SD and individual mice are shown as circles. There was a statistically significant decrease in parasite 18 S rRNA copy numbers in all peptide-immunized groups compared to adjuvant only control (one-way ANOVA log-transformed values with Tukey’s test, adjusted **P = 0.0012, *P = 0.0019, ***P = 0.0010). The difference in parasite rRNA copy number between intact and CD4 + or CD8 + T-cell-depleted mice was not statistically significant (one-way ANOVA log-transformed values with Tukey’s test, adjusted *P* = 0.8511 and *P* = 0.9967, respectively. **c** Pooled immune serum obtained post 3rd dose of T1T*/ISA 720 was passively transferred by i.v. injection to naïve recipients prior to challenge by exposure to the bites of PfPb-infected mosquitoes. Control mice received pooled sera from naïve mice or mice immunized with ISA 720 adjuvant only. Due to limited supply, only two mice received serum from mice immunized with ISA 720 adjuvant only. Parasite 18 S rRNA copy numbers in liver extracts obtained 40 h post-challenge were measured by qRT-PCR. Results shown as 18 S rRNA copy number mean ± SD with individual mice shown as closed circles. Recipients of T1T*/ISA 720 immune serum had a statistically significant decrease in parasite 18 S rRNA copy numbers when compared to recipients of naïve serum (unpaired t-test in log-transformed values, *P* = 0.0010).

The antirepeat antibodies elicited by immunization with the linear T1T* peptide were also functional in vivo (Fig. 4b). Challenge of T1T* /ISA 720 immunized C57BL/6 mice by exposure to bites of mosquitoes infected with PfPb transgenic parasites demonstrated >90% reduction of liver parasite burden when compared to adjuvant only control (adjusted *P < 0.012). Protection in the challenged mice was antibody mediated, as depletion of CD4 + or CD8 + T cells prior to challenge did not abrogate immune resistance. Similar to intact T1T* immunized mice, T-cell-depleted T1T* immunized mice had >90% reduction of parasite 18 S rRNA copy number when compared to mice immunized with ISA 720 adjuvant only. There was no significant difference in rRNA copy numbers in CD4- and CD8-depleted mice compared to intact mice (adjusted *P* = 0.8317 and *P* = 0.7685, respectively). The lack of a direct role for cell mediated immunity in resistance to challenge is consistent with our previous studies in repeat peptide-immunized mice^24^.

To confirm that in vivo sporozoite-neutralizing activity was antibody mediated, serum of mice immunized with T1T* /ISA 720 was passively transferred by intravenous injection (i.v.) into naïve recipients prior to challenge by exposure to the bites of PfPb-infected mosquitoes (Fig. 4c). The recipients of T1T*/ISA 720 immune serum had >90% reduction of parasite density in liver compared to naïve mice (*P = 0.0010), confirming that protection in the T1T* immunized mice was antibody mediated.

### MAB specific for major or minor repeats derived from peptide-immunized mice

**Fig. 5:**
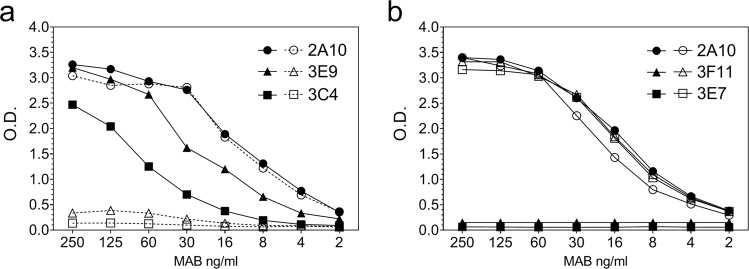
Fine specificity of MAB derived from mice immunized with linear minor or major repeat peptides. **a** MABs 3E9 and 3C4 derived from mice immunized with (T1T*) minor repeat peptide, or **b** MABs 3F11 and 3E7 derived from mice immunized with the (BT*) major repeat peptide, were tested by ELISA against wells coated with minor repeat peptide (closed symbols) or major repeat peptide (open symbols).

To further define the fine specificity of the antirepeat antibody response, MAB were derived from the mice immunized with either the T1T* minor repeat or BT* major repeat peptides. When screened by ELISA, MAB specific for either minor or major repeats could be readily obtained from the mice immunized with each immunogen (Fig. 5). MABs 3E9 and 3C4, two representative MAB from T1T* immunized mice, reacted with minor repeat (closed symbols) but not major repeats (open symbols) (Fig. 5a). Conversely, two representative MABs from BT* immunized mice, MABs 3F11 and 3E7, gave high levels of reactivity with major, but not minor repeats (Fig. 5b). MAB 2A10, cross-reacted with both minor and major repeats and confirms comparable peptide coating in each of the peptide ELISA.

**Fig. 6:**
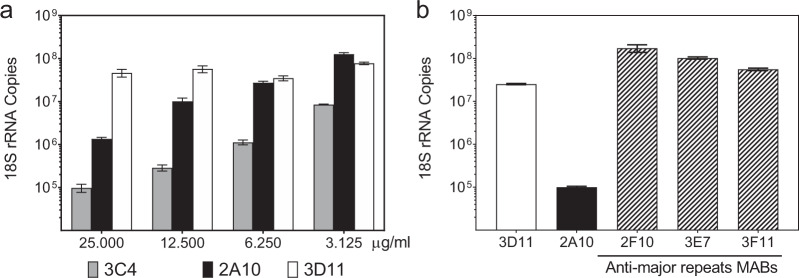
Sporozoite-neutralizing activity of MAB derived from mice immunized with minor or major repeat peptides. **a** TSNA was carried out with varying concentrations of MAB 3C4, specific for minor repeats, and MAB 2A10 that cross-reacts with minor and major repeats. MAB 3D11 specific for *P. berghei* repeats served as negative control. Results are shown as 18 S rRNA copy number mean ± SD as measured by qRT-PCR. Incubation with MAB 3C4 resulted in statistically significant lower parasite rRNA copy number at all concentrations tested when compared to MAB 3D11 (One-way ANOVA with Dunnett’s test. adjusted *P* < 0.0001). MAB 2A10 at 25 µg/ml and 12.5 µg/ml significantly decreased 18 S rRNA copy number (one-way ANOVA with Dunnett’s test, adjusted *P* < 0.0001). **b** TSNA was carried out using 12.5 µg/ml of MAB specific for major repeats (2F10, 3E7, 3F11), MAB 2A10 or MAB 3D11. Results are shown as mean ± SD parasite 18 S rRNA copy number as measured by qRT-PCR. The parasite rRNA copy numbers in cultures containing major repeat MABs were not decreased compared to MAB 3D11. As expected, the positive control MAB 2A10 reduced 18 S rRNA copy number >90% when compared to MAB 3D11 (one-way ANOVA with Dunnett’s test, adjusted *P* < 0.0001).

When tested in TSNA, the minor repeat-specific MAB 3C4 inhibited PfPb sporozoite invasion into hepatoma cells at levels comparable to or greater than MAB 2A10 derived from *P. falciparum* sporozoite-immunized mice (Fig. 6a). In contrast, several MAB derived from mice immunized with the major repeat peptide were not inhibitory at the highest concentration tested (12.5 µg/ml) (Fig. 6b). Therefore, as illustrated by these major repeat-specific MABs, high levels of antirepeat antibodies as measured by ELISA do not necessarily predict neutralizing activity against viable parasites.

## Discussion

A recent malaria vaccine milestone was reached with the WHO supported pilot implementation of a CS-based vaccine, RTS,S, in three African nations^25^. RTS,S vaccine efficacy was 30–50% in infants and children, respectively^26^, and efforts to improve CS-based vaccines are ongoing in many laboratories. While evidence for protection mediated by antirepeat antibodies has been obtained in RTS,S clinical trials^23,26^, a correlate of protective immunity for CS-based vaccines has not yet been defined. A better understanding of the antibody response to *P. falciparum* CS repeats is required to design more efficacious vaccines.

In testing of polyclonal and monoclonal antibodies, we found variation in both fine specificity and function of antibodies specific for *P. falciparum* CS repeats. MAB derived from mice immunized with *P. falciparum* sporozoites, such as MAB 2A10, reacted with both major and minor repeats, while other MAB skewed to recognition of the minor repeats (Fig. 2a). MAB specific for the minor repeats neutralized PfPb sporozoite infectivity in vitro similar to MAB 2A10 and preincubation with minor, but not major, repeat peptide decreased sporozoite-neutralizing activity (Fig. 2b, c).

Antibodies that recognized the minor CS repeats could also be elicited in C57BL/6 mice by immunization with peptide immunogens containing the T1 minor repeats (Figs. 3a, 4a). A similar skewing of antibodies to minor repeat could be elicited by T1T* peptide immunogens in BALB/c mice (Supplementary Table 1) indicating that ability to elicit antibodies to the minor repeats is not limited by genetic background.

The antibodies elicited by minor repeat peptide immunogens neutralized sporozoite infectivity in vitro in a dose-dependent manner (Fig. 3b). Consistent with in vitro results, protection in C57BL/6 mice immunized with a linear T1T* peptide was antibody mediated (Fig. 4b, c). T-cell-depleted T1T*-immunized mice had similar levels of protection as intact mice and passive transfer of immune serum from these mice protected naïve recipients following PfPb challenge. Minor repeat-specific MAB derived from T1T* immunized mice efficiently neutralized PfPb sporozoite infectivity in vitro *(*Fig. 6a). Of note, several MAB specific for the major repeats, isolated from mice immunized with BT* major repeat peptide, lacked neutralizing activity when tested in TSNA (Fig. 6b), illustrating that not all antirepeat antibodies are equally effective in neutralizing sporozoite infectivity.

The current findings demonstrate that the *P. falciparum* CS minor repeat region contains an important sporozoite-neutralizing epitope. The functional variations of the antirepeat antibody response could have potentially significant biological consequences. Vaccine efficacy would be lower if the majority of antibodies elicited by vaccines recognize only the more numerous major repeats and have minimal neutralizing capacity, as in Fig. 6b, in contrast to specifically targeting, or cross-reacting, with the minor repeats, as in Fig. 6a. The presence of protective and nonprotective epitopes within the repeats potentially provides an explanation for the paradox of why the parasite would express a highly immunogenic repeat region. As originally proposed by Anders for repeat epitopes of blood-stage antigens^27^, the more numerous major repeats may potentially serve as an immunological “smoke screen” to divert responses away from the development of inhibitory antibody responses. The design of immunogens that contain only minor repeats in the absence of major repeats, as in the T1T* peptide immunogens, may drive responses toward a higher proportion of functional minor repeat-specific antibodies and away from major repeat antibodies that are potentially non-neutralizing. Analysis of a larger panel of MAB is required to determine if the frequency of neutralizing vs non-neutralizing antibodies is increased following immunization with minor repeat peptide as compared to major repeat peptides.

A mechanism for why antibodies to minor repeats may be more protective was suggested by recent studies using human MABs derived from sporozoite-immunized malaria-naïve or African adults^28,29^. These human MAB were dual specific and bound to a unique junctional epitope within the KQPADGNPDPNANP sequence preceding the CS repeat region, as well as to NANP repeats. Passive transfer of human MAB reactive with the junctional epitope derived from *P. falciparum* sporozoite-immunized Tanzanian adults protected humanized mice against *P. falciparum* sporozoite challenge^28^. The junctional epitope is located three amino acids upstream of a previously identified proteolytic CS cleavage site in Region 1^10^. A human MAB specific for the junctional epitope blocked cleavage of *P. falciparum* CS protein^29^. MAB 2A10, which cross-reacts with both major and minor repeats, can also inhibit the proteolytic processing of CS protein^30^.

The human MABs specific for the junctional epitope recognized two distinct minimal epitopes within the junctional region, DPNANP^28^ and NPN^29^. BALB/c mice immunized with a (NPDP)_19_ peptide containing the junctional epitope, elicited high titers of antibodies specific for the junctional epitope that were poorly reactive with repeats and did not have sporozoite-neutralizing activity^28^. Additional studies by Oyen et al. have shown that dual binding of antibody to both the junctional and repeat epitopes was required for neutralizing activity^31^.

The minor repeat peptide DPNA**NPN**VDPNA**NPN**V, used as immunogen in the current studies (Fig. 1), contains two copies of the junctional epitopes identified by Kisalu et al.^29^ (indicated in bold) and Tan et al. (underlined)^28^. Whether the antibodies specific for the minor repeats, or MAB that cross-react with minor repeats such as MAB 2A10, can also recognize the junctional epitope and thus show dual specificity of binding to CS remains to be determined. Studies to examine affinity of larger panels of murine anti-T1 MAB and to identify key residues in the T1 epitope by positional scanning and generation of peptide-antibody complexes are planned.

Recent immunoglobulin gene sequencing studies have also detected a range of fine specificity of human recombinant MAB specific for CS repeats derived from volunteers immunized by exposure to *P. falciparum* sporozoites under chloroquine prophylaxis^32^. The human MAB recognized multiple epitopes within the *P. falciparum* CS repeat region, including NVDP and NANPNVDP. The current studies in sporozoite- and peptide-immunized mice are consistent with the diversity of antirepeat antibodies found in the MAB from sporozoite-immunized human volunteers and support the use of the more experimentally amendable small rodent model for analysis of antibody responses to *P. falciparum* CS repeat region relevant to the design of more efficacious human malaria vaccines.

The variable fine specificity and function of the antirepeat antibodies detected in the current studies has significant implications for the design of subunit vaccines that elicit inhibitory, rather than nonfunctional, antirepeat antibodies. Epitope-focused vaccines based on the CS minor repeat, such as the T1T* immunogen, potentially can focus the immune responses on sporozoite-neutralizing epitopes and reduce immune response to more numerous but potentially less protective major repeats. Our current studies, showing that immunization with minor repeat peptides elicits neutralizing antibodies that inhibit sporozoite infectivity in vitro and in vivo, combined with recent findings with human MABs^28,29,32^, provide a framework for the design of subunit vaccines that elicit optimal levels of neutralizing antibodies to more effectively target CS protein on *P. falciparum* sporozoites.

## Methods

### Ethics statement

Studies in mice were approved by the Institutional Animal Care and Use Committee, NYU School of Medicine (Protocol 1501081) and conducted following recommendations specified in the Guide for the Care and Use of Laboratory Animals of the National Institutes of Health.

### Peptides

For the peptide constructs, the *P. falciparum* CS major repeats were represented by the (NANP)_3_ sequence (designated “B” epitope) (Fig. 1). The 5’ minor repeat region, comprised of alternating NVDP and NANP repeats, was represented by the peptide sequence (DPNANPNVDPNANPNV). The minor repeat sequence was designated T1, as it also contains the first CS-specific CD4 + T helper cell epitope identified in sporozoite-immunized human volunteers^33^. The major or minor repeat sequences were synthesized in tandem with a malaria universal Th cell epitope, EYLNKIQNSLSTEWSPCSVT, designated T* (Fig. 1). The T* T helper cell epitope, located in the C-terminus of the *P. falciparum* CS protein (NF54 isolate), is recognized in the context of multiple murine and human class II molecules and was included in all constructs to overcome genetic restrictions^34–36^. The peptide immunogens were synthesized either as a tetrabranched construct containing the respective repeat and the T* sequences in each of four branches, or as a linear peptide using standard F-Boc peptide chemistry (AnaSpec, San Jose, CA).

### Antibodies

Fine specificity and function of antirepeat antibodies was carried out using polyclonal sera elicited by peptide immunization of C57BL/6 (H-2^b^) and BALB/c mice (H-2^d^) mice (Jackson Labs, Bar Harbor Maine). BALB/c mice were included in the initial immunogenicity studies to examine genetic restriction of humoral immunity elicited by the peptide immunogens. Since BALB/c are nonresponders to CS repeats, the peptides were synthesized to contain T*, a universal T helper cell epitope^36–39^ (Fig. 1). BALB/c are high responders to the T* T helper cell epitope, while C57BL/6 are high responders to CS repeats and the T* epitope^36,39^.

Mice were immunized subcutaneously (s.c.) at 14 day intervals with three doses of 50 µg peptide with or without various adjuvants. Adjuvants included QS21, a purified saponin derivative (Antigenics Inc., Woburn, MA); water-in-oil adjuvants, either Montanide ISA 51, a mineral oil emulsion comparable to Freund’s Incomplete Adjuvant, or ISA 720 a natural metabolizable nonmineral oil and mannide monooleate emulsifier (Seppic, Inc, Fairfield, NJ); or CpG 1826, a TLR 9 agonist comprised of synthetic oligodeoxynucleotide containing unmethylated CpG motifs (InvivoGen, San Diego, CA).

MAB 2A10, a monoclonal antibody specific for CS repeats (ATCC BEI MRA-183), generated in our laboratory from mice immunized with *P. falciparum* sporozoites^18^, was included as a standard. MAB 2A10 has been studied extensively in the analysis and development of CS subunit vaccines^40–43^. MAB 3D11, specific for *P. berghei* CS repeats^44^, was included as a negative control, as the PfPb transgenics express *P. falciparum* CS repeat region within the *P. berghei* CS protein^17^. MAB 2A10 neutralizes infectivity of PfPb sporozoites but not *P. berghei* sporozoites and, conversely, MAB 3D11 neutralizes *P. berghei*, but not PfPb sporozoites^17^.

### Serological assays

MAB fine specificity and antirepeat IgG endpoint titers in immune sera were determined by ELISA using wells coated with synthetic tetrabranched peptides representing either the major repeat (NANP)_3_ peptide or minor repeat (DPNANPNV)_2_ peptide. Two-fold dilutions of MAB or serum were tested and bound antibody was detected by reaction with HRP-labeled anti-mouse IgG (MP Biochemicals, Solon, OH) and TMB/H_2_O_2_ substrate (KPL, SeraCare; Gaithersburg, MD). Endpoints were determined as final dilution of serum giving OD > 3X BSA-coated wells. A >4-fold difference in antibody geometric mean titers was considered meaningful. Reactivity with *P. falciparum* sporozoites was determined by Indirect Immunofluorescence Assay (IFA), as in previous studies^39,45^.

In vitro studies of antibody function used a Transgenic Sporozoite Neutralization Assay (TSNA), as previously described^46–48^. In the PfPb transgenic rodent parasites, the *P. berghei* CS repeat region has been replaced with the entire *P. falciparum* CS repeat region, (NANPNVDP)_3_(NANP)_15_NVDP(NANP)_19_, as well as 26 aa of the N-terminus, NNEDNEKLRKPKHKKLKQPGDGNPDP preceding the repeat region^17^. For TSNA, MAB or polyclonal serum at various dilutions were incubated with 2 × 10^4^ PfPb sporozoites for 40 min on ice prior to addition to confluent human hepatoma HepG2 cells (ATCC HB 8065) in cRPMI (RPMI 1640 supplemented with 10%FBS, 50U Penicillin/50 µg Streptomycin, sodium pyruvate, non-essential amino acids, all from Gibco, ThermoFisher, Waltham, MA). Plates were incubated at 5% CO_2_ for 48 h, with media change at 24 h, followed by extraction of total RNA (PureLink, RNA Mini Kit, ThermoFisher, Waltham, MA). The amounts of parasite 18 S rRNA in *ea*ch culture extract was quantitated by real-time PCR (qRT-PCR) using cDNA primers specific for 18 S ribosomal RNA (rRNA)^46,49^. The parasite 18 S rRNA copy number was calculated based on a standard curve generated with known amounts of plasmid 18 S cDNA. To assess the peptide specificity of antibodies mediating sporozoite neutralization, a peptide competition TSNA was carried out in which MAB (25 µg/ml) were pre-incubated with various concentrations of either minor repeat peptide (DPNANPNV)_2_ or major repeat peptide (NANP)_3_ for one hour prior to addition of PfPb sporozoites and performance of TSNA per protocol.

For in vivo studies, peptide-immunized and naive C57BL/6 mice were challenged by exposure to the bites of 10–15 PfPb-infected mosquitoes. BALB/c mice were not used in these studies due to nonspecific resistance to *P. berghei* sporozoites^21,22^. Following sporozoite challenge, total RNA was extracted from the livers 40-h post-challenge and levels of parasites in the liver quantitated by qRT- PCR, as for in vitro TSNA. In passive transfer studies, immune serum was passively transferred to naïve recipients by intravenous injection 24 h prior to exposure to the bites of PfPb-infected mosquitoes.

Previous studies have found that >90% reduction in PfPb parasite copy number measured by qRT-PCR is associated with delayed parasitemia or sterile immunity^24,47,48^. The >90% cutoff is consistent with early studies in which i.v. injection of known numbers of sporozoites of rodent and non-human primate Plasmodium species demonstrated that a log reduction in parasite numbers results in a delay in prepatent period or absence of infection^50,51^.

### Statistical analysis

Statistical analysis was carried out using GraphPad Prism version 8.4.2. Differences between control and experimental groups were determined significant by one-way ANOVA with post-hoc Dunnett’s or Tukey’s test to correct for multiple comparisons or unpaired *t*-test. Normal distribution and equal standard deviation were corroborated. Differences were considered statistically significant at *P* < 0.05. Adjusted *P* values for multiple comparison are reported.

### Reporting summary

Further information on research design is available in the Nature Research Reporting Summary linked to this article.

## Supplementary information

Supplementary Information

Reporting Summary

## Data Availability

All data presented in the studies described here are included in this article or will be available upon request from the corresponding author.
