# Supplementary material for: Identification of a neutralizing epitope within minor repeat region of Plasmodium falciparum CS protein
Source: NPJ Vaccines. 2021 Jan 18;6:10. doi: 10.1038/s41541-020-00272-6 (PMC7813878; doi:10.1038/s41541-020-00272-6)
Supplement: Supplementary file 1 — Supplementary Information [file 41541_2020_272_MOESM1_ESM.pdf]

Supplementary Table and Figures

**Supplementary Table 1**: Fine specificity of anti-repeat antibodies elicited in BALB/c mice immunized with tetrabranched peptides containing CS major or minor repeats		
**Immunogen**[1]	**ELISA Titers**[2]	
	T1 Minor Repeats	B Major Repeats
$(T1T^*)_4$	81,920	10,240
$(BT^*)_4$	5,120	40,960

1. BALB/c mice were immunized s.c. with three doses of $(T1T^*)_4$ peptide containing minor repeats $(DPNANPNV)_2$, or $(BT^*)_4$ peptide containing major repeats $(NANP)_3$, synthesized in tandem with universal Th epitope T*.

2. IgG titers against minor or major repeat peptides measured in pooled serum obtained post 3rd dose. ELISA titers shown as highest dilution of serum giving OD against repeat peptide >3X OD BSA coated wells. A $\geq$ four-fold difference in titer was considered meaningful.

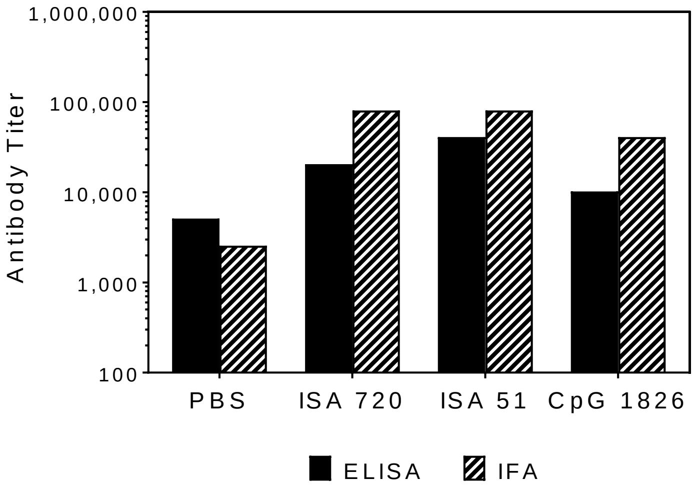

Supplementary Figure 1. Antibody responses in BALB/c mice immunized with linear peptide T1T*

BALB/c mice were immunized with three s.c. doses of linear T1T* peptide without adjuvant (PBS), or with adjuvant comprised of oil-in-water emulsions (ISA 720, ISA 51) or a TLR 9 agonist CpG. Two-fold dilutions of pooled sera were tested against tetrabranched $(T1B)_4$ repeat peptide by ELISA with endpoint titer taken as final serum dilution giving >3X OD of BSA coated wells, or by IFA using *P. falciparum* sporozoites.

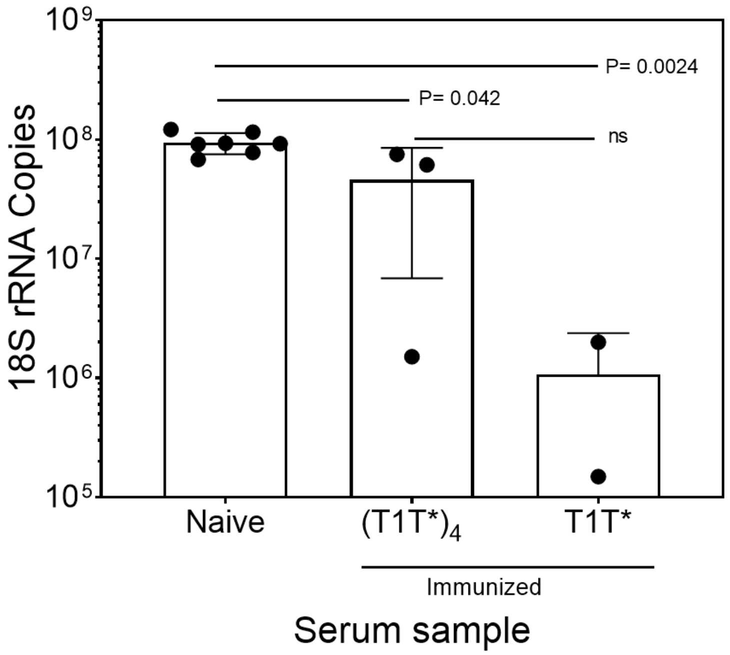

Supplementary Figure 2: Sporozoite neutralizing activity of serum derived from mice immunized with tetrabranched $(T1T^*)_4$ or linear T1T* peptides.

TSNA was used to measure neutralizing antibody elicited by tetrabranched peptide $(T1T^*)_4$ versus linear peptide T1T* formulated in ISA 720. Results shown as mean +/- SD of 18S rRNA measured by qRT-PCR. Immune sera of C57BL/6 mice immunized with either tetrabranched or linear T1T* was significantly lower when compared to naive sera (one-way ANOVA with Tukey's test, adjusted P values).
