# Supplementary material for: Identification of a neutralizing epitope within minor repeat region of Plasmodium falciparum CS protein
Source: NPJ Vaccines. 2021 Jan 18;6:10. doi: 10.1038/s41541-020-00272-6 (PMC7813878; doi:10.1038/s41541-020-00272-6)
Supplement: Supplementary file 2 — Reporting Summary [file 41541_2020_272_MOESM2_ESM.pdf]

nature research

Corresponding author(s): Calvo-Calle

Last updated by author(s): Dec 10, 2020

Reporting Summary

Nature Research wishes to improve the reproducibility of the work that we publish. This form provides structure for consistency and transparency in reporting. For further information on Nature Research policies, see our Editorial Policies and the Editorial Policy Checklist.

Statistics

For all statistical analyses, confirm that the following items are present in the figure legend, table legend, main text, or Methods section.

n/a	Confirmed	
☐	☒	The exact sample size (*n*) for each experimental group/condition, given as a discrete number and unit of measurement
☒	☐	A statement on whether measurements were taken from distinct samples or whether the same sample was measured repeatedly
☐	☒	The statistical test(s) used AND whether they are one- or two-sided<br>*Only common tests should be described solely by name; describe more complex techniques in the Methods section.*
☒	☐	A description of all covariates tested
☐	☒	A description of any assumptions or corrections, such as tests of normality and adjustment for multiple comparisons
☐	☒	A full description of the statistical parameters including central tendency (e.g. means) or other basic estimates (e.g. regression coefficient) AND variation (e.g. standard deviation) or associated estimates of uncertainty (e.g. confidence intervals)
☒	☐	For null hypothesis testing, the test statistic (e.g. *F*, *t*, *r*) with confidence intervals, effect sizes, degrees of freedom and *P* value noted<br>*Give P values as exact values whenever suitable.*
☒	☐	For Bayesian analysis, information on the choice of priors and Markov chain Monte Carlo settings
☒	☐	For hierarchical and complex designs, identification of the appropriate level for tests and full reporting of outcomes
☒	☐	Estimates of effect sizes (e.g. Cohen's *d*, Pearson's *r*), indicating how they were calculated

Our web collection on statistics for biologists contains articles on many of the points above.

Software and code

Policy information about availability of computer code

Data collection	No software was used for data collection
Data analysis	GraphPad Prism version 8.4.2.<br>The parasite 18S rRNA copy number was calculated based on a standard curve generated with known amounts of plasmid 18S cDNA calculated in Excel

For manuscripts utilizing custom algorithms or software that are central to the research but not yet described in published literature, software must be made available to editors and reviewers. We strongly encourage code deposition in a community repository (e.g. GitHub). See the Nature Research guidelines for submitting code & software for further information.

Data

Policy information about availability of data

All manuscripts must include a data availability statement. This statement should provide the following information, where applicable:

- Accession codes, unique identifiers, or web links for publicly available datasets
- A list of figures that have associated raw data
- A description of any restrictions on data availability

Provide your data availability statement here.

Field-specific reporting

Please select the one below that is the best fit for your research. If you are not sure, read the appropriate sections before making your selection.

☒ Life sciences ☐ Behavioural & social sciences ☐ Ecological, evolutionary & environmental sciences

For a reference copy of the document with all sections, see nature.com/documents/nr-reporting-summary-flat.pdf

Life sciences study design

All studies must disclose on these points even when the disclosure is negative.

Sample size	Each immunization group included 2-3 mice during the immunogenicity screening and subsequent studies up to 4 mice for pairwise comparisons between experimental and control groups.
Data exclusions	No data points were excluded
Replication	Reported studies are from representative experiments. Studies always included the same Positive and negative controls.
Randomization	Mice were randomly assigned to groups for immunization
Blinding	Blinding was not done in this study

Reporting for specific materials, systems and methods

We require information from authors about some types of materials, experimental systems and methods used in many studies. Here, indicate whether each material, system or method listed is relevant to your study. If you are not sure if a list item applies to your research, read the appropriate section before selecting a response.

Materials & experimental systems

n/a	Involved in the study	
☐	☐	Antibodies
☐	☒	Eukaryotic cell lines
☒	☐	Palaeontology and archaeology
☐	☒	Animals and other organisms
☒	☐	Human research participants
☒	☐	Clinical data
☒	☐	Dual use research of concern

Methods

n/a	Involved in the study	
☒	☐	ChIP-seq
☒	☐	Flow cytometry
☒	☐	MRI-based neuroimaging

Antibodies

Antibodies used	Anti- P. falciparum CS 2A10 (MRA-183A); Anti P. berghei 3D11 (MRA-100); anti-mouse CD4 GK1.5 (ATCC TIB-207); anti-mouse CD8 MAb 2.43)
Validation	Antibody were validated with intended target and control antigens.

Eukaryotic cell lines

Policy information about cell lines

Cell line source(s)	HepG2 cells from ATCC HB 8065.
Authentication	No re-authentication performed.
Mycoplasma contamination	Cells tested for Mycoplasma using commercial kits.
Commonly misidentified lines (See ICLAC register)	*Name any commonly misidentified cell lines used in the study and provide a rationale for their use.*

Animals and other organisms

Policy information about studies involving animals; ARRIVE guidelines recommended for reporting animal research

Laboratory animals	Six to eight weeks old C57BL/6 and BALB/c female mice from The Jackson Laboratory, Bar Harbor , ME
Wild animals	None
Field-collected samples	NA
Ethics oversight	Studies in mice were approved by the Institutional Animal Care and Use Committee, NYU School of Medicine (Protocol 1501081)

Note that full information on the approval of the study protocol must also be provided in the manuscript.
